# Within- and cross-mental health disorder correlations in husband-and-wife pairs

**DOI:** 10.1186/s12888-022-04335-x

**Published:** 2022-12-05

**Authors:** Ray M. Merrill

**Affiliations:** Department of Public Health, College of Life Sciences, Brigham Young University, 2063 Life Sciences Building, Provo, UT 84602 USA

**Keywords:** Age, Employee health, Mental disorders, Sleep disorders, Spouse

## Abstract

**Background:**

Mental health disorders can adversely affect relationships and are heritable. Yet, there is a high prevalence of mental illness in spouses and partners of those with mental illness. This study will assess within- and cross-mental health disorder correlations in husband-and-wife pairs.

**Methods:**

A cross-sectional study design was employed using medical claims data from the Deseret Mutual Benefit Administrators (DMBA), linked to demographic information from employee eligibility files, 2020. Analyses involved 21,027 contract holders aged 18–64 (68.6% male, 31.4% female), with sub-analyses on 16,543 married individuals. Summary statistics, as well as rates, and rate ratios adjusted for age, sex, and dependent child status were calculated to describe the data.

**Results:**

The rate of stress is 19.2%, anxiety is 26.4%, and depression is 23.6% in spouses of contract holders with the same respective disorders. Rates of stress, anxiety, and depression in a spouse are greatest if the contract holder has schizophrenia. Rates of mental illness in wives of male contract holders experiencing mental health disorders tend to be greater than the rates of mental illness in husbands of female contract holders experiencing mental health disorders. Rates of stress, anxiety, and depression in spouses of contract holders tend to be 2–3 times greater when the contract holder has a mental health disorder, after adjusting for the contract holder’s age, sex, dependent child status, and difference in age within husband-and-wife pairs. However, differences in the magnitude of observed associations vary. The rate of a spouse having stress is 5.5 times greater if the contract holder has schizophrenia (vs. does not have schizophrenia), whereas the rate of a spouse having stress is 1.4 times greater if the contract holder has sleep apnea (vs. does not have sleep apnea).

**Conclusion:**

Mental health disorders in spouses of contract holders are greater if the contract holder has a mental health disorder, more so when the contract holder has more serious mental illness. Both within- and cross-mental disorder correlations exist. These results have implications on relationship quality and the mental health of offspring.

## Background

It is well known in couples’ research that when one individual has mental illness, it can negatively impact their companion’s mental health. A study of 9740 couples in Norway during 1995–1997 found that spouses of persons with mental health disorders scored significantly higher on symptoms of anxiety and depression compared with spouses of persons without mental health disorders [1]. Symptoms associated with mental health disorders, such as limited emotional availability, difficulty in following through on household tasks, job loss, and lack of desire to socialize, can put strain on the spouse’s or partner’s mental health [2]. In addition, codependence may ensue, increasing the risk of behaviors like manipulation, name-calling, and other harmful dynamics, which can increase the risk of a mental health disorder in a spouse or partner. For example, in a 1998 study of 105 depressed women in the United States, depression and codependency were strongly correlated [3].

In addition to the possibility of a mental health disorder developing as a result of the initial mental illness in a spouse or partner, mental illness may exist in a relationship because individuals tend to deliberately or unconsciously choose relationships with individuals similar to themselves [4, 5]. In a longitudinal study of 12,010 seniors aged 65 years or older, assessed between 1993 and 2010, a concordance was found between wife and husband depressive symptoms, which tended to increase with age [6]. The authors of that research suggested that this may be because individuals often choose spouses similar to themselves, with similar social backgrounds. Shared environments (e.g., financial pressures, similar risk behaviors and illness) and emotional contagion may also play a role.

While disentangling the relative effect of an individual’s mental health on their companion’s mental health may be difficult, the fact that associations exist has implications on relationship stability and children. A multinational survey involving 12 countries (*n* = 30,729) published in 2011 found that married individuals with mental health disorders were more likely to divorce [7]. In a meta-analysis of 33 studies with 3863 offspring of parents with schizophrenia, bipolar disorder, or depression, published in 2012, researchers found that children of these parents were at increased risk for a range of mental health disorders, with 32% (95% CI 24–42%) developing schizophrenia, bipolar disorder, or depression by early adulthood [8].

In a study of 707,263 Swedish residents, conducted in 2014–2015, positive correlations of mental health disorders between companions were identified [9]. Within-mental disorder correlations were marginally higher than cross-mental disorder correlations. A mental health disorder in an individual was generally associated with a 2 to 3-fold increase in their companion’s odds of having the same or another mental health disorder. The companion’s odds of having the same or another mental health disorder were greatest when the individual had ADHD, ASD, or schizophrenia.

Association between an individual’s mental health disorder and that of their spouse or partner may be confounded or modified by age, sex, dependent child status, and age differences within couples. The purpose of the current study was to identify positive correlations of mental health disorders between contract holders and their spouses based on healthcare claims in a large insurance database. Within-mental health disorder and cross-mental health disorder correlations will be assessed. Confounding and modifying influences of age, sex, dependent child status, and age differences in spouses will also be considered.

## Methods

### Study population

The current study involves individuals insured by the Deseret Mutual Benefit Administrator (DMBA) company in 2020. The company (established in 1970) provides health insurance and retirement income to contract holders and their family members. The company is owned and managed by the Church of Jesus Christ of Latter-day Saints. In the study year, enrollees represented 26% contract holders, 21% spouses, and 53% dependent children. Contract holders work in the Church education system, seminaries, and institutes (36%); as manual labors (33%); in other companies (11%); and in other capacities (20%). They reside in Utah (74%), Idaho (9%), Pacific states (9%), and other American states (8%). Employee retention from 2019 to 2020 was 92% (80% in ages 18–29, 95% in ages 30–64, and 76% in ages 65 or older).

### Data collection

Automated healthcare claims data used in this study were retrieved for the year 2020 and linked to the employee file, which contains demographic and employment information. Claims and employee records were linked using a unique employee identifying number. Following the linkage, the database was de-identified according to Health Insurance Portability and Accountability Act (HIPAA) guidelines. The authors institutional review board gave the study exemption status based on regulatory and institutional criteria.

Primary outcome variables involved mental health indicators. International Classification of Diseases, Tenth Revision, Clinical Modification (ICD-10-CM) codes were used to classify mental health disorders [10]. Specific mental health disorders considered were anxiety (F40, F1), depression (F32, F33), stress (F43), attention deficit hyperactivity disorder (ADHD) (F90), bipolar disorder (F31), obsessive-compulsive disorder (OCD) (F42), schizophrenia (F20–F29), and autism F(84.0). Stress consists of a reaction to severe stress and adjustment disorder. It is applicable to an acute crisis reaction, an acute reaction to stress, a combat and operational stress reaction, combat fatigue, a crisis state, and psychic shock.

The rate of a mental health disorder consists of the number of enrollees filing a claim for that disorder divided by the number of enrollees. If multiple claims were filed for a given disorder, it was only counted once in the numerator of the rate calculation. However, an individual could contribute to more than one type of mental health condition. Because only more serious mental health disorders tend to seek treatment, rates of mental health disorders reported in this study will not reflect some of the moderate or less severe cases. Hence, the rates will tend to be lower than obtained from cross-sectional surveys.

Other variables considered in this study include age, sex, marital status (married, single), and dependent child status (yes, no). Among married contract holders, the age difference between each husband-and-wife pair is calculated. This is done by subtracting the husband’s age from the wife’s age. In the study, age is treated as both a continuous variable and as a categorical variable (18–29, 30–39, 40–49, 50–64).

The study does not include contract holders aged 65 years or older because a high percentage of these individuals drop DMBA insurance as they pick-up Medicare. Hence, the current study is based on 21,027 contract holders aged 18–64 in 2020, and their spouses. A sub-analysis is performed on 16,543 married employees.

### Statistical techniques

Counts, percentages, means, and standard deviations were used to describe the data. Rates of mental health disorders (per 100) were calculated according to age, sex, marital status, and dependent child status. Rates may or may not reflect first time cases. Rate ratios and corresponding 95% confidence intervals were used to assess associations between contract holder’s mental health disorders (stress, anxiety, depression ADHD, bipolar disorder, OCD, schizophrenia, and autism, and insomnia and sleep apnea, which correspond with mental illness) and age, sex, marital status, and dependent child status. Further, for married contract holders, their spouses’ mental health status was assessed according to their own mental health status, based on rate ratios adjusted for age, sex, and dependent child status. Finally, mean differences in age between husband-and-wife pairs, with the latter subtracted from the former, were compared according to the contract holder’s status of stress, anxiety, and depression, adjusting for age, sex, and dependent children. Interactions were considered in the models. Two-sided tests of significance were used, based on the 0.05 level. Statistical analyses were derived from Statistical Analysis System (SAS) software, version 9.1 (SAS Institute Inc., Cary, NC, USA, 2003).

## Results

### Description of mental health among contract holders

The distribution of age, sex, marital status, and dependent child status is shown for contract holders in Table 1. Contract holders are more likely to be men than women, in the age range 40–64, married, and have dependent children. Mean age is similar between sexes (M = 46.5 [SD = 11.1] for men and 46.2 [SD = 12.4] for women). Men are more likely to be married than women (89.0% vs. 56.1%, *p* < .0001) and are more likely to have dependent children (77.3% vs. 43.4%, *p* < .0001).

**Table 1:** Mental health disorders among contract holders according to age, sex, marital status, and dependent children status, 2020

			Mental Health Disorders								Sleep Disorders	
			Stress	Anxiety	Depression	ADHD	Bipolar Disorder	OCD	Schizo-phrenia	Autism	Insomnia	Sleep Apnea
	No.	%	Rate per 100	Rate per 100	Rate per 100	Rate per 100	Rate per 100	Rate per 100	Rate per 100	Rate per 100	Rate per 100	Rate per 100
Overall			2.27	9.49	8.52	2.01	0.58	0.04	0.11	0.03	2.13	10.13
Age (years)												
18–29	2084	9.91	2.21	10.99	8.30	2.21	0.72	0.86	0.05	0.00	0.67	1.63
30–39	4521	21.5	2.43	10.31	8.32	2.46	0.42	0.58	0.11	0.02	1.02	5.26
40–49	5643	26.84	2.60	10.24	9.36	2.29	0.60	0.44	0.12	0.05	2.25	9.11
50–64	8779	41.75	1.99	8.22	8.13	1.55	0.63	0.17	0.11	0.03	2.96	15.30
*p*-value			0.185	<.001	0.703	0.001	0.6256	<.001	0.551	0.463	<.001	<.001
Sex												
Men	14,419	68.57	1.66	7.77	6.89	2.21	0.58	0.43	0.10	0.04	2.03	11.73
Women	6608	31.43	3.60	13.24	12.08	1.57	0.59	0.33	0.12	0.02	2.35	6.63
*p*-value			<.001	<.001	<.001	0.002	0.946	0.300	0.729	0.329	0.135	<.001
Marital Status												
Married	16,543	78.68	2.01	8.91	7.75	2.07	0.56	0.38	0.08	0.04	2.16	11.15
Single	4484	21.32	3.23	11.62	11.35	1.76	0.67	0.47	0.22	0.02	1.98	6.33
*p*-value			<.001	<.001	<.001	0.187	0.405	0.410	0.010	0.649	0.460	<.001
Dependent Children												
No	7018	33.38	2.38	10.60	9.43	1.47	0.76	0.46	0.16	0.01	2.08	9.18
Yes	14,009	66.62	2.22	8.93	8.06	2.28	0.50	0.37	0.09	0.04	2.15	10.60
*p*-value			0.464	<.001	0.001	<.001	0.022	0.358	0.142	0.284	0.746	0.001

*p*-values are based on the Chi-square test

Approximately 16.5% (*n* = 3474) of the contract holders filed claims for one or more of the mental health disorders listed (not including the sleep disorders). The rate of filing one or more claims for each of the selected mental health disorders is shown in Table 1. The rate of anxiety, ADHD, and OCD significantly decreases with age, whereas the rate of insomnia and sleep apnea increases with age. Comparisons of the rates between men and women, married and singles, and no dependent children and dependent children are also presented. Women compared with men have higher rates of stress, anxiety, and depression, but lower rates of ADHD and sleep apnea. Singles compared with married have higher rates of stress, anxiety, depression, and schizophrenia, but lower rates of sleep apnea. Contract holders with dependent children have lower rates of anxiety and bipolar disorder but higher rates of ADHD and sleep apnea. The rate ratio involving marital status (single vs. married) and anxiety (yes vs. no) become insignificant after adjusting for age, sex, and dependent children (data not shown). Rate ratios involving dependent children (yes vs. no) and anxiety (yes vs. no), dependent children and depression (yes vs. no), and dependent children (yes vs. no) and sleep apnea (yes vs. no) also become insignificant after adjusting for age, sex, and marital status (data not shown).

### Rate of contract Holder’s spouses mental illness according to their own mental illness

Rates of mental illness in spouses of contract holders who have a given mental illness are shown in Table 2. The rate of spouses having the same type of mental illness (or insomnia or sleep apnea, which are associated with mental illness) as the contract holders is greatest for stress, anxiety, depression, and sleep apnea. Rates of spousal stress are greatest if the contract holder has stress or schizophrenia; rates of spousal anxiety are greatest if the contract holder has schizophrenia or autism; and rates of spousal depression are greatest if the contract holder has schizophrenia. In general, spousal rates of anxiety and depression associated with contract holder mental illnesses are greater than spousal rates of stress associated with contract holder mental illnesses. The rates of mental illness in wives of male contract holders experiencing mental illness tend to be greater than the rate of mental illness in husbands of female contract holders experiencing mental illness. For example, the wife of a male contract holder with stress is 2.3 times as likely to experience stress than the husband of a female contract holder with stress.

**Table 2:** Rates of mental health disorders in spouses of contract holders according to the contract holder having a selected mental health disorder, 2020

Mental Health Disorder of the Contract Holder		Same Mental Health Disorder in Spouse		Stress in Spouse		Anxiety in Spouse		Depression in Spouse	
	No.	No.	Rate per 100	No.	Rate per 100	No.	Rate per 100	No.	Rate per 100
Both Men and Women									
Stress	333	64	19.22	64	19.22	78	23.42	91	27.33
Anxiety	1474	389	26.39	93	6.31	389	26.39	308	20.90
Depression	1282	302	23.56	79	6.16	293	22.85	302	23.56
ADHD	343	37	10.79	22	6.41	65	18.95	70	20.41
Bipolar Disorder	93	5	5.38	6	6.45	27	29.03	24	25.81
OCD	63	0	0.00	4	6.35	16	25.40	10	15.87
Schizophrenia	13	0	0.00	2	15.38	6	46.15	6	46.15
Autism	6	0	0.00	0	0.00	3	50.00	1	16.67
Insomnia	358	34	9.50	16	4.47	74	20.67	62	17.32
Sleep Apnea	1845	348	18.9	67	3.63	305	16.53	297	16.10
Men									
Stress	219	52	23.74	52	23.74	60	27.40	70	31.96
Anxiety	1007	297	29.49	67	6.65	297	29.49	227	22.54
Depression	886	235	26.52	62	7.00	241	27.20	235	26.52
ADHD	288	29	10.07	21	7.29	61	21.18	61	21.18
Bipolar Disorder	78	3	3.85	6	7.69	23	29.49	21	26.92
OCD	55	0	0.00	4	7.27	16	29.09	9	16.36
Schizophrenia	11	0	0.00	2	18.18	6	54.55	6	54.55
Autism	6	0	0.00	0	0.00	3	50.00	1	16.67
Insomnia	271	31	11.44	13	4.80	64	23.62	52	19.19
Sleep Apnea	1592	256	16.08	66	4.15	278	17.46	269	16.90
Women									
Stress	114	12	10.53	12	10.53	18	15.79	21	18.42
Anxiety	467	92	19.70	26	5.57	92	19.70	81	17.34
Depression	396	67	16.92	17	4.29	52	13.13	67	16.92
ADHD	55	8	14.55	1	1.82	4	7.27	9	16.36
Bipolar Disorder	15	2	13.33	0	0.00	4	26.67	3	20.00
OCD	8	0	0.00	0	0.00	0	0.00	1	12.50
Schizophrenia	2	0	0.00	0	0.00	0	0.00	0	0.00
Autism	0	0	0.00	0	0	0.00	0	0	0.00
Insomnia	87	3	3.45	3	3.45	10	11.49	10	11.49
Sleep Apnea	253	92	36.36	1	0.40	27	10.67	28	11.07

Applies to 16,543 married employees in DMBA, 2020

Spouses have significantly higher rates of stress, anxiety, and depression when the contract holder has any of the specific mental health disorders (vs. has not), with only a few exceptions (Table 3). These exceptions involve the association between the contract holder’s OCD status and their spouses stress status and depression status, and between the contract holder’s autism status and their spouse’s depression status. Statistical insignificance here is likely due to small numbers. The rate of stress in a spouse is most strongly associated with the contract holder having stress or schizophrenia, and least strongly associated with the contract holder having a sleep disorder. The rate of anxiety in a spouse is most strongly associated with the contract holder having anxiety, schizophrenia, or autism, and least strongly associated with the contract holder having ADHD or a sleeping disorder. Finally, the rate of depression in a spouse is most strongly associated with the contract holder having depression, stress, or schizophrenia.

**Table 3:** Rates of spousal stress, anxiety, or depression according to contract holder’s mental health status, 2020

Contract Holder’s Mental Health Disorder (yes vs. no)	Stress (yes vs. no)			Anxiety (yes vs. no)			Depression (yes vs. no)		
	Rate Ratio^a^	95% LCL^a^	95% UCL^a^	Rate Ratio^a^	95% LCL^a^	95% UCL^a^	Rate Ratio^a^	95% LCL^a^	95% UCL^a^
Stress	8.52	6.70	10.85	2.09	1.71	2.54	2.69	2.25	3.22
Anxiety	2.83	2.26	3.54	2.61	2.37	2.88	2.20	1.97	2.46
Depression	2.66	2.10	3.37	2.15	1.93	2.40	2.48	2.22	2.77
ADHD	2.43	1.61	3.69	1.52	1.22	1.89	1.84	1.49	2.28
Bipolar Disorder	2.39	1.09	5.25	2.37	1.72	3.27	2.33	1.64	3.29
Schizophrenia	5.47	1.53	19.65	3.57	2.03	6.26	4.01	2.27	7.09
OCD	2.26	0.87	5.86	1.93	1.26	2.95	1.41	0.79	2.49
Autism				3.88	1.72	8.72	1.42	0.24	8.52
Insomnia	1.74	1.07	2.85	1.84	1.50	2.26	1.64	1.30	2.06
Sleep Apnea	1.43	1.10	1.86	1.45	1.29	1.62	1.54	1.37	1.73

*ADHD* Attention Deficit Hyperactivity Disorder, *OCD* Obsessive Compulsive Disorder

^a^Adjusted for age, sex, and dependent children

### Mental illness according to age differences between husband-and-wife pairs

Differences in age between husband-and-wife pairs range from − 25.2 to 30.6, with men being 1.84 years older (SD = 3.49), on average (*p* < .0001). Mean age differences increase for contract holders in the older age groups (1.14 [SD = 2.69] for ages 18–29, 1.38 [SD = 3.07] for ages 30–39, 1.81 [SD = 3.43] for ages 40–49, and 2.18 [SD = 3.77] for ages 50–64, *p* < .0001), is greater in women than men (2.07 [SD = 4.27] vs. 1.77 [SD = 3.22], *p* < .0001), and is also greater for those with dependent children compared with no dependent children (1.92 [SD = 3.34] vs. 1.53 [SD = 4.02], *p* < .0001).

If the husband is younger than his wife, the contract holder is 1.29 (1.02–1.62) times as likely to experience stress (1.53 [95% CI 1.16–2.03] times as likely if the contract holder is male and 0.92 [95% CI 0.61–1.40] if the contract holder is female), after adjusting for age, sex, and dependent child status. There is no significant relationship between the age difference variable and the contract holder’s rate of anxiety in the adjusted model, either overall or separately for men or women. If the husband is younger than his wife, the contract holder is 1.12 (1.00–1.26) times as likely to experience depression (1.16 [95% CI 1.01–1.34] if the contract holder is male and 1.03 [95% CI 0.84–1.27] if the contract holder is female), after adjusting for age, sex, and dependent child status. The rate of stress, anxiety, and depression in contract holders is further assessed according to the husband-and-wife age difference being at least 2 years and by sex, as shown in Table 4. The results are similar for stress and depression, but the rate of anxiety is also significantly greater in male contract holders if he is at least 2 years older than his wife.

**Table 4:** Rates of stress, anxiety, and depression in contract holders according to the husband-and-wife age difference and sex

Age Difference			Stress			Anxiety			Depression		
	No.	%	Rate Ratio^a^	95% LCL^a^	95% UCL^a^	Rate Ratio^a^	95% LCL^a^	95% UCL^a^	Rate Ratio^a^	95% LCL^a^	95% UCL^a^
Husband at least 2 years younger than his wife	1385	8.37	1.53	1.08	2.16	1.05	0.88	1.26	1.19	0.99	1.43
Within 2 years	7604	45.97	1.00	–	–	1.00	–	–	1.00	–	–
Husband is at least 2 year older than his wife	7554	45.66	1.16	0.92	1.45	1.09	0.98	1.21	1.02	0.91	1.14
Male Contract Holders											
Husband at least 2 years younger than his wife	958	7.46	1.77	1.15	2.72	1.15	0.92	1.45	1.26	1.00	1.59
Within 2 years	6068	47.27	1.00	–	–	1.00	–	–	1.00	–	–
Husband is at least 2 year older than his wife	5810	45.26	1.06	0.80	1.41	1.13	1.00	1.28	1.01	0.88	1.15
Female Contract Holders											
Husband at least 2 years younger than his wife	427	11.52	1.25	0.68	2.27	0.91	0.68	1.22	1.10	0.81	1.48
Within 2 years	1536	41.44	1.00	–	–	1.00	–	–	1.00	–	–
Husband is at least 2 year older than his wife	1744	47.05	1.34	0.90	1.99	0.99	0.83	1.19	1.04	0.85	1.28

Age difference refers to the husband’s age minus the wife’s age

^a^Adjusted for age, sex, year, and dependent children

Finally, the rate ratios in Table 3 were further adjusted for the husband-and-wife paired age difference variable. The addition of this variable did not significantly contribute to any of the models, nor did it modify any of the rate ratios in the table.

## Discussion

This study identified positive correlations of mental health disorders between contract holders and their spouses based on healthcare claims in a large insurance database. Significant within-mental health disorder and cross-mental health disorder correlations were identified, after adjusting for age, sex, dependent child status, and husband-and-wife age differences. Results are consistent with previous research [9]. The current study also assessed the potential modifying influence of age, sex, dependent child status, and age differences in spouses.

### Description of study population

A description of the study population showed rates of mental illness to be lower than generally seen in cross-sectional surveys. Mental health statistics in the United States indicate that in 2020, 21% (52.9 million) of adults aged 18 years or older had some form of mental illness, but 5.6% (14.2 million) had serious mental illness [11]. The current study found 16.5% of contract holders filed claims for one or more of the mental health disorders considered in this study (not including the sleep disorders). The lower percent is expected, given that cases were considered for a single year and only those with more serious conditions were likely to file a claim.

Anxiety, ADHD, and OCD rates decreased with age. In a consistent manner, the mental health statistics in the United States 2020 survey found that younger adults aged 18–25 years had the highest prevalence of any mental illness compared with adults aged 26–49 years (25.3%) and aged 50 or more years (14.5%)  [11]. As for ADHD, for example, this disorder is typically diagnosed in childhood and wanes with age [12]. In addition, a small number of individuals may outgrow ADHD [13].

The study found that for stress, anxiety, and depression rates were higher for women than men but for ADHD lower for women. The mental health statistics in the United States 2020 survey found mental illness was greater in women (25.8%) than men (15.8%) [11]. The finding of higher rates of stress in women is consistent as reported in the literature [14]. Higher stress in women, in turn, may contribute to higher anxiety and depression in women [15], with women consistently showing higher anxiety and depression in other studies [16, 17]. Lower rates of ADHD in women is also consistent with findings reported in other research [18].

The study found that stress, anxiety, depression, and schizophrenia rates were higher for singles. In a similar fashion, in a cross-sectional household survey conducted in 15 countries from the World Health Organization (WHO) World Mental Health survey initiative (*n* = 34,493), published in 2010, marriage (vs. never married) was associated with lower risk of first onset of several mental disorders in both males and females [19].

Having children is associated with various stressors, especially when the child has a disability or a mental health disorder [20, 21]. After adjusting for age, sex, and marital status, the study found that there was no increased rate of stress, anxiety, or depression for those with a dependent child or children. For ADHD the rate was higher in those with a dependent child or children but for bipolar disorder the rate was lower. The positive association between having a dependent child or children and having ADHD is unclear and requires further research. The negative association between having a dependent child or children and bipolar disorder is consistent with individuals with bipolar disorder being less likely to have children.

### Within- and cross-mental health disorder correlations

Within-mental disorder correlations are greater for stress, anxiety, depression, and sleep apnea and lower for the other, perhaps more serious mental health disorders. This is consistent with those with more serious mental health disorders being less likely to enter or stay in marriage. It may be that they have greater difficulty with social-emotional reciprocity, back-and-forth conversation, initiating or responding to social interaction, and reduced sharing of interests, emotions, or affects [22].

Cross-mental health disorder correlations were greatest between spousal stress, anxiety, and depression and contract holder schizophrenia. The long-term seriousness of schizophrenia, relapses, auditory hallucinations, unemployment, and economic slide are major stressors that contribute to challenges in marital relationships and increased risks for anxiety and depression [23]. A common difficulty with schizophrenia treatment is that antipsychotic medications can have major side effects [24]. Another common difficulty with schizophrenia treatment is non-adherence, or failure to take medications as prescribed [25].

Spousal rates of anxiety and depression associated with contract holder mental illnesses are greater (up to over 4-times so for some disorders) than spousal rates of stress associated with contract holder mental illnesses. Difference in a spouse’s stress, anxiety, and depression when the contract holder has stress is the smallest. Hence, more serious mental health conditions in the contract holder are associated with comparatively greater rates of anxiety and depression. An exception is with sleep disorders, where if the contract holder has a sleep disorder, the spousal rates of anxiety and depression are noticeably higher than the rate of stress. Although it is well known that sleep apnea can cause depression in the individual with the disorder [26], and that many of the risk factors for sleep apnea overlap with the symptoms of depression [27], the current study shows that it has a noticeable influence on a spouse. The results further show that stress, anxiety, and depression rates are noticeably greater for spouses of male contract holders than spouses of female contract holders. A similar result was observed for insomnia and other mental health disorders.

Rates of stress, anxiety, and depression in spouses of contract holders tend to be 2–3 times greater when the contract holder has a mental health disorder, as consistent with another study [9]. Differences in the magnitude of observed associations vary. For example, the rate of a spouse having stress is 5.5 times greater if the contract holder has schizophrenia versus does not have schizophrenia, whereas the rate of a spouse having stress is 1.4 times greater if the contract holder has sleep apnea versus does not have sleep apnea. The weaker correlation with non-psychiatric conditions like a sleep disorder is also consistent with former research [9].

Rates of mental health disorders were described according to age differences in husband-and-wife pairs. Husbands were 1.84 years older, on average, than their wives. The average age difference in married couples is a little lower than in the United States as a whole, where, on average, men are about 2.3 years older than their spouse [28]. In modern cultures, there is a tendency for men to seek younger companions and for women to seek older companions. Women often marry older men who are better financially able to care for them, whereas men tend to be attracted to younger women, in part for childbearing reasons and attractiveness [29–32].

### Husband-and wife age differences impact on mental health

The results showed that when the husband was younger than his wife, the husband had greater stress and depression, but the wife did not. In addition, when the husband was at least 2 years older than the wife (vs. within 2 years of age), the husband had greater anxiety, but the wife did not. In general, women were not significantly affected by age differences in terms of stress, anxiety, and depression. However, this result may change if seniors were included in the study. In a study conducted in Korea among 2881 couples in 2006, 30,033 couples in 2008, and 2772 couples in 2010, and 2711 couples in 2012 who were at least 45 years of age at baseline, for every 1–2 extra years in age difference between husbands and wives estimated severity of depressive symptoms significantly increased [33].

### Limitations and potential bias

1. Because the study data are based on enrollment and medical claims from all husband-wife pairs in the DMBA database during 2020, selection bias is not a potential threat to the validity of the study.
2. A mental health diagnosis will not lead to losing insurance, so selective under-reporting of mental health claims for this reason is unlikely. However, it may be that some individuals experiencing a mental health disorder will not be identified, if a claim is not filed for treating the disorder.
3. Incorrect diagnosis and treatment of mental health are possible. The available data did not allow the level of under- or over-reporting of mental health to be determined.
4. The fact that less serious mental health disorders are not likely captured in the claims database may limit generalizability.
5. The data reflects a single year such that causal directions cannot be evaluated.
6. The data is limited in that it is unknown whether the spouses of the contract holders are employed outside the home or taking care of children, nor is the education level of the enrollees known or the type of housing in which they reside.

## Conclusion

Both within- and cross-mental health disorder correlations exist. Rates of mental health disorders in spouses of contract holders are greater if the contract holder also has a mental health disorder. Although the rates tend to range from 2 to 3 greater, they are even higher for more serious mental illness like schizophrenia and lower for sleep disorders (which may be more treatable and less likely to pose communication problems). The significantly positive within- and cross-mental disorder correlations are likely the result of two forces: symptoms associated with mental health disorders (e.g., communication challenges, long-term seriousness, and treatments) putting strain on the spouse’s mental health and individuals choosing a companion like themselves, with similar social backgrounds. The results may be useful to healthcare workers as they counsel couples and talk about potential psychiatric consequences for offspring.

## Data Availability

The datasets generated and analyzed during the current study are not publicly available due to confidentiality restrictions but are available in a de-identified and aggregated format from the corresponding author on reasonable request.

## Abbreviations

ADHD: Attention Deficit Hyperactivity Disorder
DMBA: Deseret Mutual Benefit Administrators
HIPAA: Health Insurance Portability and Accountability Act
ICD-10-CM: International Classification of Diseases, Tenth Revision, Clinical Modification
OCD: Obsessive compulsive disorder

## References

[1] Idstad M, Ash H, Tambs K (2010). Mental disorder and caregiver burden in spouses: the Nord-Trøndelag health study. BMC Public Health.

[2] Mokoena AG, Poggenpoel M, Myburgh C, Temane A (2019). Lived experiences of couples in a relationship where one partner is diagnosed with a mental illness. Curationis..

[3] Hughes-Hammer C, Martsolf DS, Zeller RA (1998). Depression and codependency in women. Arch Psychiatr Nurs.

[4] Maes HH, Neale MC, Kendler KS, Hewitt JK, Silberg JL, Foley DL, Meyer JM, Rutter M, Simonoff E, Pickles A, Eaves LJ (1998). Assortative mating for major psychiatric diagnoses in two population-based samples. Psychol Med.

[5] Van den Broucke S, Vandereycken W (1994). Ill health in spouses of psychiatric patients: cause or consequence?. J Psychosoc Nurs Ment Health Serv.

[6] Pradeep N, Sutin AR. Spouses and depressive symptoms in older adulthood. Sci Rep. 2015:5. 10.1038/srep08594. 10.1038/srep08594 PMC4341217 25716455

[7] Breslau J, Miller E, Jin R (2011). A multinational study of mental disorders, marriage, and divorce. Acta Psychiatr Scand.

[8] Rasic D, Hajek T, Alda M, Uher R (2014). Risk of mental illness in offspring of parents with schizophrenia, bipolar disorder, and major depressive disorder: a meta-analysis of family high-risk studies. Schizophr Bull.

[9] Nordsletten AE, Larsson H, Crowley JJ, Almqvist C, Lichtenstein P, Mataix-Cols D (2016). Patterns of nonrandom mating within and across 11 major psychiatric disorders. JAMA Psychiatry.

[10] World Health Organization (2004). ICD-10: international statistical classification of diseases and related health problems: tenth revision.

[11] Substance Abuse and Mental Health Services Administration (SAMHSA). 2020 National Survey on Drug Use and Health. https://www.samhsa.gov/data/release/2020-national-survey-drug-use-and-health-nsduh-releases. Accessed 8 Aug 2022.

[12] Biederman J, Mick E, Faraone SV (2000). Age-dependent decline of symptoms of attention deficit hyperactivity disorder: impact of remission definition and symptom type. Am J Psychiatry.

[13] Anixt JS, Vaughn AJ, Powe NR, Lipkin PH (2016). Adolescent perceptions of outgrowing childhood attention-deficit hyperactivity disorder: relationship to symptoms and quality of life. J Dev Behav Pediatr.

[14] Verma R, Balhara YP, Gupta CS (2011). Gender differences in stress response: role of developmental and biological determinants. Ind Psychiatry J.

[15] Khan S, Khan RA (2017). Chronic stress leads to anxiety and depression. Ann Psychiatry Ment Health.

[16] Bahrami F, Yousefi N (2011). Females are more anxious than males: a metacognitive perspective. Iran J Psychiatry Behav Sci.

[17] Albert PR (2015). Why is depression more prevalent in women?. J Psychiatry Neurosci.

[18] Rucklidge JJ (2010). Gender differences in attention-deficit/hyperactivity disorder. Psychiatr Clin North Am.

[19] Scott KM, Wells JE, Angermeyer M (2010). Gender and the relationship between marital status and first onset of mood, anxiety and substance use disorders. Psychol Med.

[20] Merrill RM, Smith A, Schenk CC (2021). Mental health of families with autism spectrum disorder: a systematic review. OSP J Pediatr.

[21] Marquis SM, McGrail K, Hayes M (2020). Mental health of parents of children with a developmental disability in British Columbia, Canada. J Epidemiol Community Health.

[22] Koegel L, Ashbaugh K, Nawab A, Koegel R (2016). Improving verbal empathetic communication for adults with autism spectrum disorder. J Autism Dev Disord.

[23] Thara R, Srinivasan TN (1997). Outcome of marriage in schizophrenia. Soc Psychiatry Psychiatr Epidemiol.

[24] Dilbaz N (2015). New targets for the management of schizophrenia. Klinik Psikofarmakol.

[25] Viveiros CP, Tatar CR, dos Santos DVD, Stefanello S, Nisihara R (2020). Evaluation of nonadherence to treatment among patients with schizophrenia attending psychosocial care centers in the south region of Brazil. Trends Psychiatry Psychother.

[26] Jackson ML, Tolson J, Bartlett D, Berlowitz DJ, Varma P, Barnes M (2019). Clinical depression in untreated obstructive sleep apnea: examining predictors and a meta-analysis of prevalence rates. Sleep Med.

[27] Cai L, Xu L, Wei L, Sun Y, Chen W (2017). Evaluation of the risk factors of depressive disorders comorbid with obstructive sleep apnea. Neuropsychiatr Dis Treat.

[28] World Marriage (2013). United Nations, Department of Economic and Social Affairs, Population Division.

[29] Buss DM, Schmitt DP (1993). Sexual strategies theory: an evolutionary perspective on human mating. Psychol Rev.

[30] Young JA, Critelli J, W, Keith KW. (2005). Male age preferences for short-term and long-term mating. Sex Evol Gender.

[31] Buss DM (1989). Sex differences in human mate preferences: evolutionary hypotheses tested in 37 cultures. J Behav Brain Sci.

[32] Schwarz S, Hassebrauck M (2012). Sex and age differences in mate-selection preferences. Hum Nat.

[33] Kim J, Park E, Lee SG (2015). The impact of age differences in couples on depressive symptoms: evidence from the Korean longitudinal study of aging (2006-2012). BMC Psychiatry.

